# miRNA-142-3p aggravates hydrogen peroxide-induced human umbilical vein endothelial cell premature senescence by targeting SIRT1

**DOI:** 10.1042/BSR20231511

**Published:** 2024-05-15

**Authors:** Pengfei Tong, Jingke Zhang, Shuang Liu, Jiyang An, Gehan Jing, Laifeng Ma, Ruihua Wang, Zhengfeng Wang

**Affiliations:** 1 Department of Neurosurgery, The Third People’s Hospital of Henan Province, Zhengzhou 450006, China; 2 Department of Neurosurgery, The First Affiliated Hospital of Zhengzhou University, Zhengzhou, 450052, China; 3 Department of Nuclear Medicine, The First Affiliated Hospital of Zhengzhou University, Zhengzhou, 450052, China

**Keywords:** endothelial cells, miRNA-142-3p, oxidative stress, Senescence, sirtuins, stroke

## Abstract

Vascular endothelial cell premature senescence plays an important part in stroke. Many microRNAs (miRNAs) are known to be involved in the pathological process of vascular endothelial cell premature senescence. The present study aimed to investigate the mechanism of hydrogen peroxide (H_2_O_2_)-induced premature senescence in human umbilical vein endothelial cells (HUVECs) and effect of miR-142-3p on hydrogen peroxide (H_2_O_2_)-induced premature senescence. HUVECs were exposed to H_2_O_2_ to establish a model premature senescence in endothelial cells. CCK-8 assay was performed to detect cell viability. Senescence-associated β-galactosidase staining assay and senescence-related proteins p16 and p21 were used to detect changes in the degree of cell senescence. RT-qPCR and Western blot were conducted to measure mRNA and protein levels, respectively. The scratch wound-healing assay, transwell assay, and EdU assay were performed to evaluate the ability of migration and proliferation, respectively. miRNA-142-3p and silencing information regulator 2 related enzyme 1 (SIRT1) binding was verified using Targetscan software and a dual-luciferase assay. We found that miRNA-142-3p is abnormally up-regulated in HUVECs treated with H_2_O_2_. Functionally, miRNA-142-3p inhibition may mitigate the degree of HUVEC senescence and improve HUVEC migration and proliferation. Mechanistically, SIRT1 was validated to be targeted by miRNA-142-3p in HUVECs. Moreover, SIRT1 inhibition reversed the effects of miRNA-142-3p inhibition on senescent HUVECs exposed to H_2_O_2_. To our knowledge, this is the first study to show that miRNA-142-3p ameliorates H_2_O_2_-induced HUVECs premature senescence by targeting SIRT1 and may shed light on the role of the miR-142-3p/SIRT1 axis in stroke treatment.

## Introduction

Stroke has long been a major disease threatening national health, with a high incidence, a high recurrence rate, a high disability rate, and a high mortality rate [1]. Thanks to improved medical care, including early diagnosis, risk factor management, and medical treatment, stroke mortality has been reduced in the past decade [2]. Despite these advances stroke treatment still faces considerable challenges, which may be addressed with a better understanding of the pathological changes that occur in stroke. For example, it is known that dysregulation of cerebrovascular homeostasis is caused by endothelial dysfunction and that this plays an important role in determining stroke [3,4].

Endothelial cells form the inner layer or intima of the vascular wall and function by responding to hormones, neurotransmitters, and physiological stimuli in order to maintain homeostasis [5]. Senescence of vascular endothelial cells can lead to vascular dysfunction and trigger cerebrovascular diseases [6,7]. Cell senescence, first proposed by Hayflick, L, is defined as a state of irreversible cell cycle arrest, which is closely related to aging [8,9]. It is typically characterized by the cessation of cell division and termination of the passage under stress [7]. There are two types of cellular senescence: replicative senescence induced by endogenous stimulation and premature senescence caused by exogenous stress [10]. Exogenous stress conditions mainly include chemoradiotherapy, peroxide, metabolic wastes, telomere damage (caused by continuous cell proliferation), etc [10,11]. Oxidative stress-induced premature senescence is a widely used in vitro model of cell senescence, among which the common irritants include hydrogen peroxide (H_2_O_2_) and D-galactose [7,12]. The degree of cumulative damage experienced by the cells determines whether senescence, programmed cell death, or necrosis occurs [9,12]. Sublethal stress accelerates the development of a senescent cell phenotype, which is associated with various cellular markers. These key senescence hallmarks include senescence-associated β-galactosidase staining, senescence-associated cyclins p53, p21, and p16, γ-H2AX and p38 mitogen-activated protein kinase in the DNA damage pathway, and senescence-associated secretory phenotype (SASP) [10,11]. Exploring the molecular mechanisms involved in the premature senescence of vascular endothelial cells in association with cerebrovascular diseases may provide new methodologies for the prevention and treatment of stroke.

MicroRNAs (miRNAs) are a class of small single-stranded non-coding RNAs, which are involved in the post-transcriptional regulation of crucial genes in many diseases [13]. Mature miRNAs, which are approximately 20–24 nucleotides in length, bind to the 3′-untranslated region (3′UTR) of target mRNAs, leading to mRNA translation inhibition and degradation [13–15]. Increasing evidence supports the idea that dysregulated microRNAs are involved in stroke [14,16]. For example, Kang Du et al. demonstrated that inhibition of miR-191 promotes angiogenesis by up-regulating vascular endothelial zinc finger 1 (VEZF1) after acute ischemic stroke [17]. Lei Huang et al. reported that knockdown of miR-210 expression can ameliorate brain injury after ischemic stroke in mice by inhibiting the pro-inflammatory response [18]. David L Bernstein et al. proposed that miR-98 lessens the degree of injury of the blood-brain barrier that is induced by endothelial dysfunction and improves locomotor impairment after stroke in mice [19]. miRNAs may also play a role in endothelial cell senescence. For example, Zhibo Wang et al. reported that inhibition of miR-217 ameliorates endothelial cell senescence by regulating the SIRT1/P53 signaling pathway [20], and Kensuke Toyama et al. proposed that miR-30a-5p, miR-181a-5p, and miR-30a-3p are closely related to vascular endothelial cell senescence [21].

miRNA-142-3p is located on chromosome 17q22 and has been shown to play an important role in the development of stroke [22]. miRNA-142-3p reduces high glucose-induced renal tubular epithelial cell injury via biorientation of chromosomes in cell division 1 (BOD1) expression [23]. miRNA-142-3p overexpression in a model of high myopia inhibits collagen I expression in human scleral fibroblasts via TGF-β1 [24]. However, little research has been performed on the role of miRNA-142-3p in hydrogen peroxide-induced endothelial cell senescence. Therefore, this study established a stable and reliable model of human umbilical vein endothelial cell senescence induced by hydrogen peroxide and investigated the miRNA-142-3p mechanism of action on senescent human umbilical vein endothelial cells.

## Methods

### Cell culture

Normal primary human umbilical vein endothelial cells (HUVECs) were obtained from American Tissue Culture Collection (ATCC; PCS-100-010™, U.S.A.). The cells were cultured in endothelial cell medium (ECM; Sciencell, U.S.A.) in a moisturizing incubator with 5% CO_2_ at 37°C. The cells were passaged when the cell density reached 80–90%. To induce an endothelial cell premature senescence model, HUVECs at 70% confluence and passages 4-7 were exposed to H_2_O_2_ (0, 25, 50, 100, 200, and 400 μM) in the ECM medium without fetal bovine serum for 1 h and incubated with ECM medium for another 24 h.

### Cell transfection

For the miRNA transfection, miRNA-142-3p inhibitor and its negative control (NC) inhibitor was synthesized by Sangon Biotech (Shanghai). The sense strand of si-Sirt1 was 5′-AGAGUUGCCACCCACACCUUU. Transfection of anti-miRNA-142-3p (UCCAUAAAGUAGGAAACACUACA) and anti-miR-NC (CAGUACUUUUGUGUAGUACAA) was performed according to the Lipofectamine® 3000 transfection reagent Invitrogen protocol (L3000015, ThermoFisher, U.S.A.). In brief, HUVECs (2 × 10^5^ cells/well) were cultured in 6-well plates until the cells reached 60–70% confluence. Lipofectamine 3000 reagent was diluted in a serum-free medium and miRNA-142-3p inhibitor (100 nmol/L) and a corresponding NC were added and incubated with the cells for 15 min at room temperature. After the complex was incubated with the cells for 6 h, the complete ECM medium was replaced and the culture was continued for 48 h at 37°C with 5% CO_2_.

### Quantitative real-time PCR (RT-qPCR) analysis

Total RNA was obtained using TRIzol reagent (Invitrogen, Carlsbad, CA, U.S.A.), according to the manufacturer’s instructions. A NanoDrop One instrument (ND-One- W, ThermoFisher, U.S.A.) was used to detect the total RNA concentrations and purity. The Mir-X miRNA First-Strand Synthesis Kit (Takara, Tokyo, Japan) was used for cDNA synthesis, and miRNA levels were quantified using a Mir-X miRNA qRT-PCR TB Green Kit (Takara) (Table 1). mRNA levels of target genes were quantified using TB Green® Premix Ex Taq™ II (Tli RNaseH Plus) (Takara). U6 and GAPDH were used as an internal control for miRNA and mRNA, respectively. Each RNA amplification was performed in triplicate, and the gene expression levels were calculated using the 2^−ΔΔCt^ calculated method. Experiments were independently conducted three times.

**Table 1:** Primer sequences

Gene symbol	Sequence (5′-3′)
miRNA-142-3p	Forward (F) TGCTGCTGTGTAGTGTTTCCTACT
	Reverse (R) TATGGTTGTTCACGACTCCTTCAC
SIRT1	F TGTGTCATAGGTTAGGTGGTGA
	R AGCCAATTCTTTTTGTGTTCGTG
U6	F CGCTTCGGCAGCACATATAC
	R TTCACGAATTTGCGTGTCATC
GAPDH	F CATGAGAAGTATGACAACAGCCT
	R AGTCCTTCCACGATACCAAAGT

### Cell counting kit-8 (CCK-8) assay

HUVEC viability was evaluated by CCK-8 (DOJINDO, Japan) assay. Briefly, HUVECs (5 × 10^3^ cells/well) were placed into 96-well plates. Following overnight adhesion, cells were treated with varying concentrations of H_2_O_2_ (0, 25, 50, 100, 200, 400 μM) for 1 h and incubated with complete ECM medium for another 24 h at 37°C. Then, 10 μl CCK-8 solution was added to each well, and cells were cultured for another 2 h at 37°C. Finally, the optical density of HUVECs was detected at 450 nm using a spectrophotometer (ThermoFisher Scientific, Waltham, MA, U.S.A.). The experiment was independently conducted three times.

### Senescence-associated β-galactosidase staining assay

A senescence-associated β-galactosidase (SA-β-gal) staining kit (Beyotime Biotechnology, China) was used to identify HUVEC senescence. Briefly, treated cells that were plated in 6-well plates were rinsed with PBS and fixed with β-galactosidase staining fixative solution for 15 min at room temperature. Cell were then rinsed with PBS and incubated overnight with β-galactosidase staining working solution at 37°C without CO_2_. An inverted microscope (Nikon, Tokyo, Japan) was used to calculate the percentage of SA-β-gal positive cells per field of view. Four fields of view were counted for each treatment group.

### Scratch wound-healing assay

HUVECs migration was evaluated by the scratch wound-healing assay. In brief, HUVECs (2 × 10^5^ cells/well) were cultured in 6-well plates, transfected with vectors for 6 h, and cell senescence was induced with 100 μM H_2_O_2_ for 1 h. After the cell reached 90% confluence, the monolayer HUVECs were scratched using a sterile 200 pipette tip and rinsed with warm PBS to remove cellular debris. The cells were photographed at 0 h and 12 h. The migration ability of HUVECs was expressed as the ratio of cell migration area. Migration area (%) = (A0 − A1)/A0 × 100, where A0 represented the wound area of 0 h and A1 represented the remaining wound area after 12 h. All images were captured and analyzed by ImageJ at the metering point.

### Transwell assay

A transwell assay was performed to evaluate the infiltrative ability of HUVECs using a 24-well transwell chamber (Corning Inc.). After 6 h of miRNA-142-3p transfection, HUVECs (1 × 10^5^ cells/well) were seeded in the upper chamber and supplemented with serum-free medium. The lower chamber was filled with complete medium with 10% fetal bovine serum to create a nutrient concentration gradient that promoted cell migration into the lower chamber. Following incubation for 24 h at 37°C, the filtrated cells were fixed with 4% paraformaldehyde for 10 minutes and stained with 0.1% Crystal Violet for 20 min at room temperature. An inverted microscope was used to calculate the number of cells per field of view. Four fields of view were counted in each treatment group.

### EdU staining assay

The proliferative ability of HUVECs was evaluated using a BeyoClick™ EdU Cell Proliferation Kit with Alexa Fluor 488 (Beyotime, Shanghai, China). Briefly, transfected HUVECs cultured in 96-well plates were incubated with 10 μM EdU buffer for 2 h at 37°C. After that, cells were fixed with 4% paraformaldehyde for 15 min at room temperature, incubated with permeable fluid for 15 min, and then stained with ‘Click Additive Solution’ for 30 min in the dark. Subsequently, cell nuclei were stained using Hoechst 33,342 (Beyotime, Shanghai, China). Finally, cells were photographed using an inverted fluorescent microscope. The proliferative ability of cells was expressed as the ratio of EdU-positive to negative cells (green). The counting function in Adobe Photoshop 2021 (version 22.3.0, Adobe™) software was used to calculate the ratio of EdU-positive cells. Four fields of view were counted in each treatment group.

### Bioinformatics prediction

miRNA-142-3p biological target genes were predicted using an internationally recognized predictive website, targetscan7.2 (https://www.targetscan.org/vert_72/).

### Dual-luciferase reporter assay

Fragments of the 3′UTR of SIRT1, containing the predicted binding site of miRNA-142-3p, and the corresponding mutated sequences were synthesized and cloned into the pmirGLO vector Dial-luciferase miRNA Target Expression Vector (Promega, U.S.A.) to construct the SIRT1-wild type (SIRT1-WT) and SIRT1-mutant type (SIRT1-MUT) reporter vectors. These vectors were co-transfected into 29-3T cells with a miRNA-142-3p mimic or NC-mimic. After 48 h of transfection, luciferase activity was detected using a dual-luciferase reporter assay system (Promega, U.S.A.).

### Western blot analysis

Total protein was extracted from HUVECs using the Radio Immunoprecipitation Assay buffer (NCM Biotech) containing protease and phosphatase inhibitors, according to the manufacturer’s instructions. The BCA protein assay kit (Epizyme Shanghai, China) was used to determine the protein concentration in protein extracts. The extracts were boiled at 100°C for 15 min to denature proteins. An equal amount of the total protein sample was separated in each sample using sodium dodecyl sulfate-polyacrylamide gel electrophoresis (SDS-PAGE), and proteins were transferred onto a polyvinylidene fluoride (PVDF) membrane. Subsequently, a protein-free rapid blocking buffer (Epizyme Shanghai, China) was used to block the PVDF membranes for 20 min at room temperature. Membranes were then incubated with primary antibodies overnight at 4°C. Primary antibodies included the rabbit monoclonal antibody against SIRT1 (1:1000, 2496, CST), rabbit polyclonal antibody against p21 (1:1000, 381102, ZENBIO), rabbit polyclonal antibody against p16-INK4a (1:1000, 380963, ZENBIO), and the rabbit polyclonal antibody against GAPDH (1:5000, ABL1021, Abbkine). The secondary antibody (Goat Anti-Rabbit IgG, HRP) was purchased from Abbkine Biotch. After being washed with tris-buffered saline containing Tween®20 (TBST), The membranes were incubated with corresponding horseradish peroxidase (HRP)-conjugated goat anti-rabbit (1:50000, A21020, Abbkine) secondary antibodies for 1 h at room temperature. Super enhanced chemiluminescence (ECL) plus (Uelandy Biotech) was used to detect protein bands, and the gray value of the bands was quantified using ImageJ software (version 5.0, Bio-Rad, Hercules, CA, U.S.A.).

### Statistical analysis

The statistical analysis was conducted using GraphPad prism version 8.02 for Windows (GraphPad Software Inc. San Diego, CA, U.S.A.). All data are presented as mean ± standard deviation (SD). Significant differences between two groups was determined using Student’s *t*-test, and comparisons of more than two groups were performed using one-way analysis of variance. Differences were considered statistically significant when the *p* value was less than 0.05. All experiments were conducted three times independently.

## Results

### H_2_O_2_ reduces cell viability and promotes premature senescence in HUVECs

**Figure 1:**
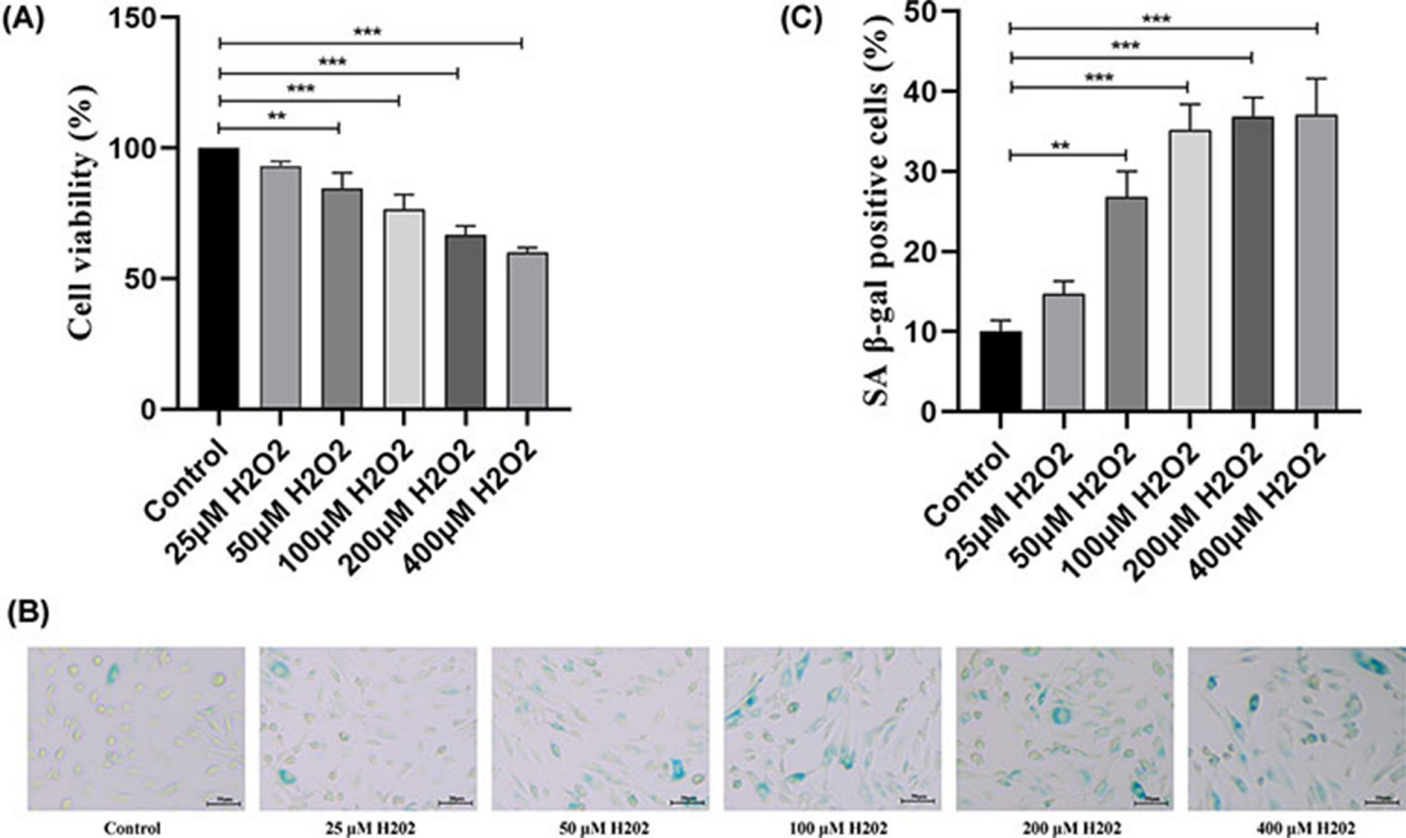
H_2_O_2_ reduces cell viability and promotes premature senescence in HUVECs (**A**) Different concentrations of H_2_O_2_ (0, 25, 50, 100, 200, and 400 μM) were applied to cultured HUVECs for 1 h. A CCK-8 assay was used to evaluate cell viability. (**B,C**) Representation and statistics of cell premature senescence in different H_2_O_2_-treated groups. Senescent cells become larger and stained blue. **P<0.01, ***P<0.001 vs. control; HUVEC, human umbilical vein endothelial cell.

To observe the effect of H_2_O_2_ on HUVEC viability, HUVECs were incubated with various concentrations of H_2_O_2_ (0, 25, 50, 100, 200, and 400 μM) for 1 h. CCK-8 assay results showed that H_2_O_2_ treatment decreased HUVEC viability in a concentration-dependent manner (Figure 1A). After incubation with the above mentioned H_2_O_2_ concentrations, a SA-β-gal staining assay was performed to study the degree of premature cellular senescence. As indicated in Figure 1B,C, increasing H_2_O_2_ concentration increased the percentage of SA-β-gal staining positive cells (morphologically large, flat, and blue), and the degree of cell premature senescence was more advanced with higher H_2_O_2_ concentrations.

### H_2_O_2_ accelerates premature cellular senescence and inhibits cell proliferation in HUVECs

**Figure 2:**
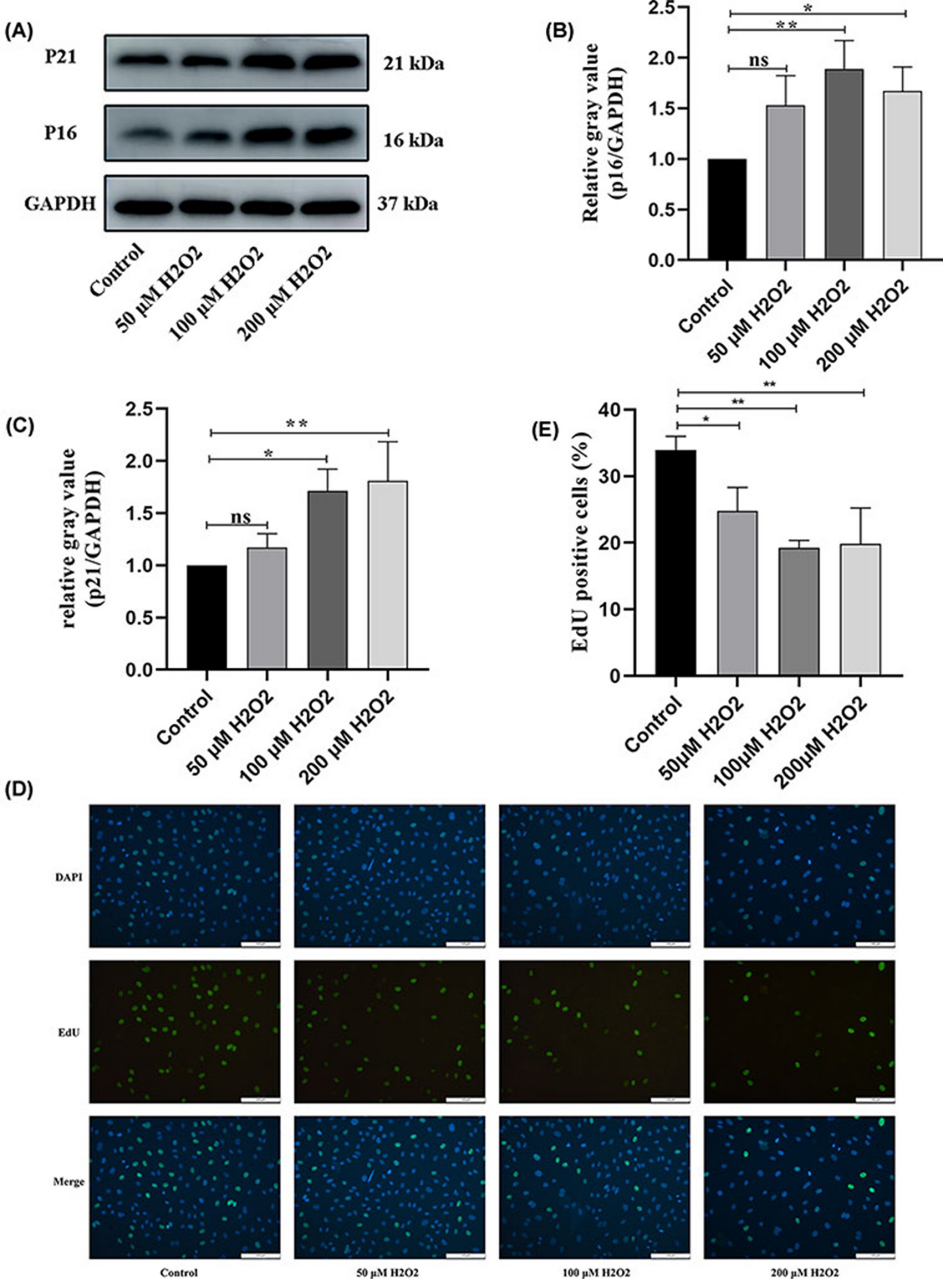
H_2_O_2_ accelerates cellular premature and inhibits cell proliferation in HUVECs (**A**) Determination of p16 and p21 protein expression by Western Blotting. (**B,C**) Densitometric quantification of p16 and p21 protein expression based on Western Blot assays. (**D,E**) Representation and statistics of cell proliferation in different H_2_O_2_ treated groups. *P<0.05, **P<0.01 vs Control, HUVEC, human umbilical vein endothelial cell; ns: no significance.

The level of senescence-associated protein expression (p21 and p16) was detected in the control and treatment groups. As shown in the Western blot (Figure 2A–C), compared with the control group, the expression levels of p16 and p21 proteins in the treatment group increased with increasing H_2_O_2_ concentration, with the 100 μM group having the highest expression levels. The effect of H_2_O_2_ on cell proliferation was evaluated by EdU assay. The result of the EdU assay demonstrated that H_2_O_2_ decreased EdU-positive cells in a dose-dependent manner (Figure 2D,E). In addition, we added 100 μM H_2_O_2_ to treat HUVECs for 1 h and then continued incubation for 24 h and 7 days. The expression levels of senescence-related proteins p16 and p21 were more significant after 7 days of cell culture compared with 24 h (Supplementary Figure SA–C). The results of SA-β-gal staining were consistent with the results (Supplementary Figure SD–E). However, this study focuses more on cellular senescence caused by early oxidative stress. According to the above results, 100 μM H_2_O_2_ incubation of HUVECs for 1 h followed by continued incubation for 24 h is the most appropriate H_2_O_2_ concentration and treatment time to establish the model of cell premature senescence.

### Relative expression of miR-142-3p and its effect on HUVEC viability

**Figure 3:**
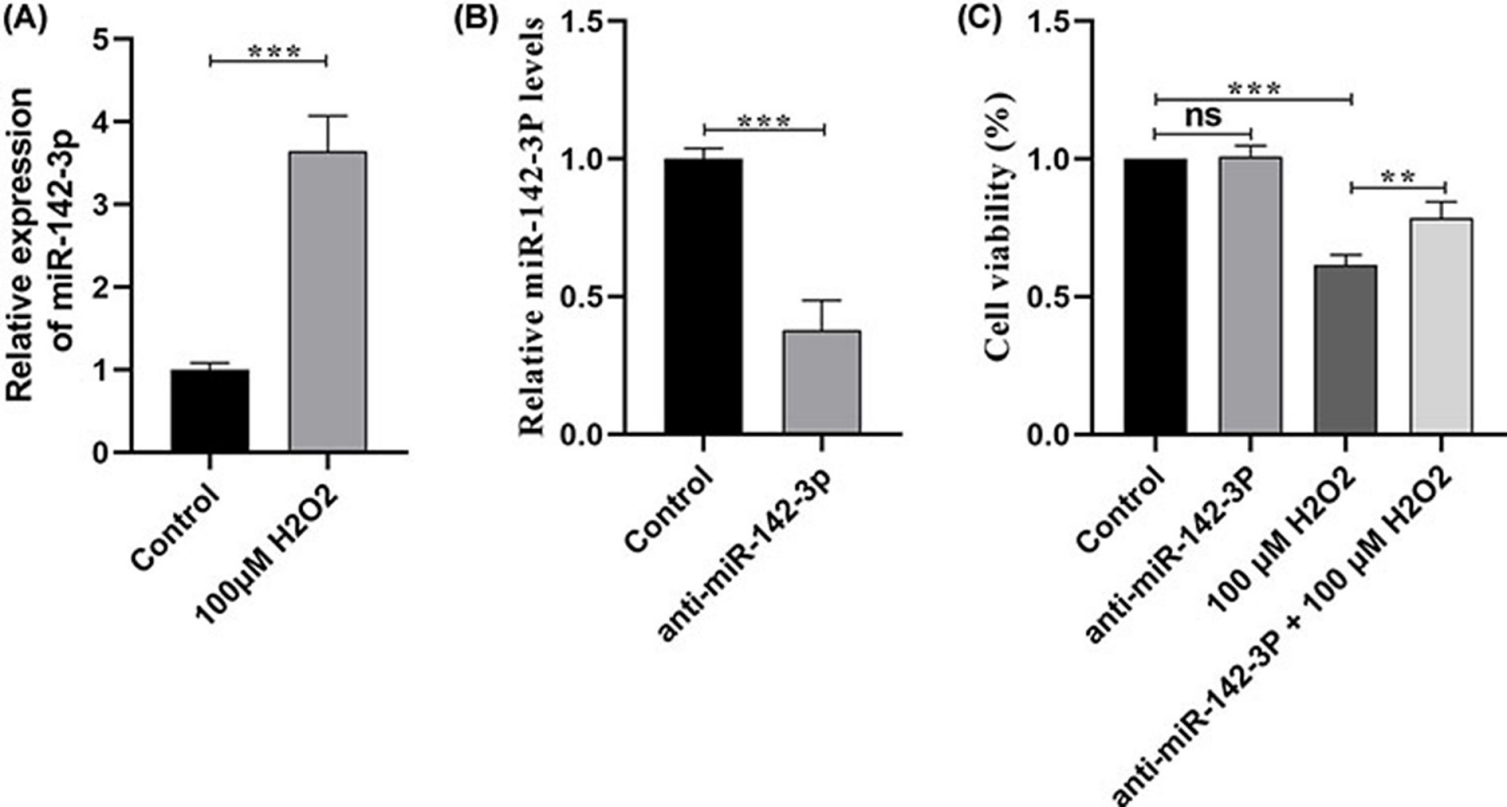
Relative expression of miR-142-3p and its effect on the cell viability of HUVECs (**A**) miR-142-3p expression levels in control and 100 μM H_2_O_2_, revealed by RT-qPCR. (**B**) The relative expression level of miR-142-3p after knocking out miR-142-3p. (**C**) Cell viability of HUVECs between different treatment groups. **P<0.01, ***P<0.001, HUVEC, human umbilical vein endothelial cell; ns: no significance.

In a previous study, miRNA-142-3p was down-regulated in peritoneal macrophages of aged mice [25], therefore, we determined the expression of miRNA-142-3p in premature senescent HUVECs induced by H_2_O_2_. RT-qPCR results showed that the expression of miRNA-142-3p was significantly up-regulated in the premature senescent HUVECs compared with the control group (Figure 3A). Afterward, to clarify the role of miRNA-142-3p in premature senescent HUVECs, the HUVECs were transfected with anti-miRNA-142-3p. The expression of miRNA-142-3p was significantly inhibited in the inhibitor group compared to the control group, as demonstrated by RT-q PCR (Figure 3B). Furthermore, the cell viability of HUVECs treated with miRNA-142-3p was examined. The CCK-8 assay showed that inhibiting miRNA-142-3p expression counteracted the cell viability decrease in HUVECs treated with H_2_O_2_.

### Knockdown of miRNA-142-3p mitigated the degree of premature senescence in H_2_O_2-_treated HUVECs

**Figure 4:**
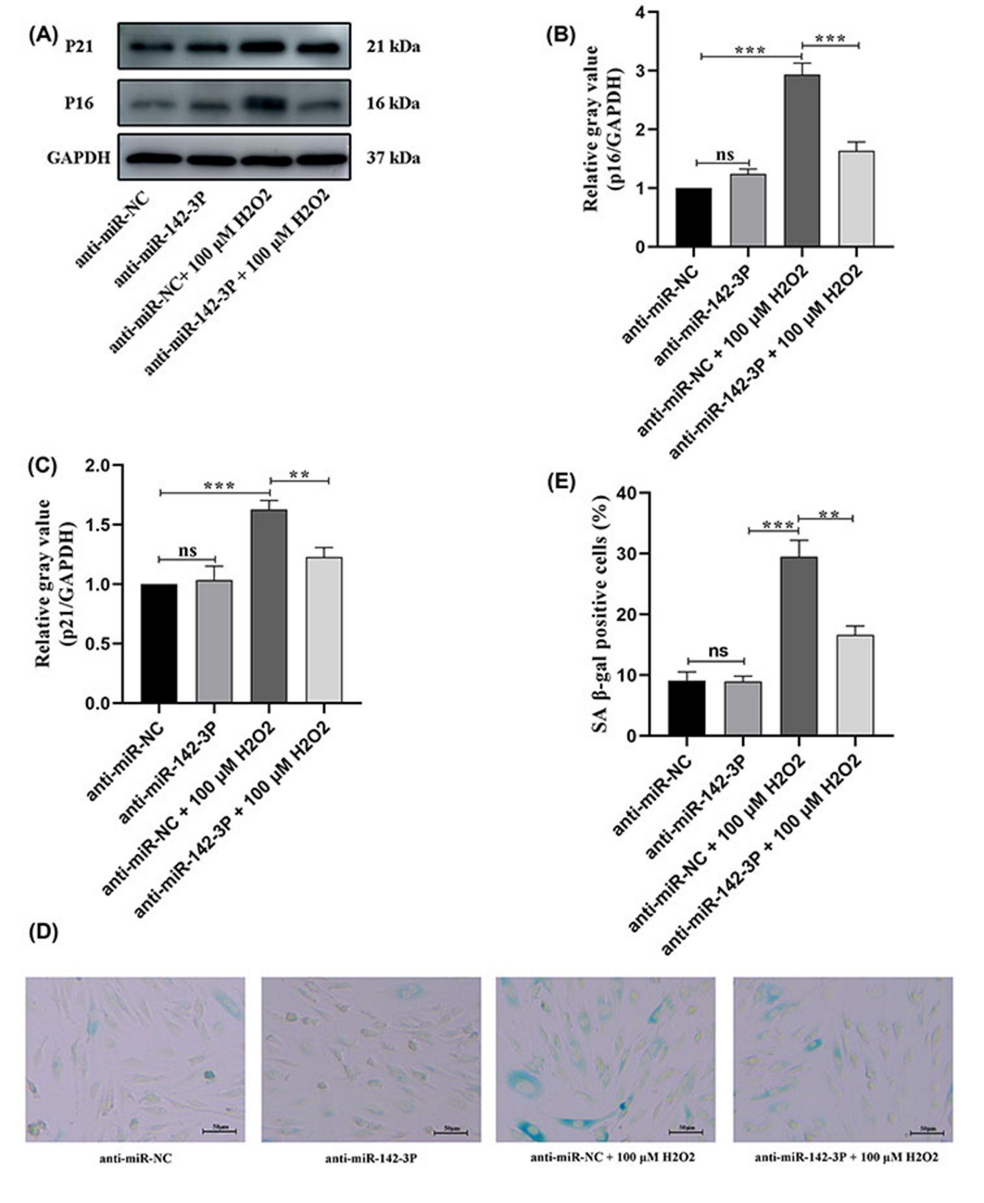
Knockdown of miR-142-3p mitigated the degree of premature senescent HUVECs induced by H_2_O_2_ (**A–C**) Representation and statistics of p16 and p21 protein relative expression in HUVECs with different miR-142-3p gene modifications. (**D,E**) Representation and statistics of cell proliferation in HUVECs with different miR-142-3p gene modifications. **P<0.01, ***P<0.001, HUVEC, human umbilical vein endothelial cell; ns: no significance.

To clarify the role of miRNA-142-3p in premature senescence in HUVECs, anti-miRNA-142-3p- and anti-miR-NC-transfected HUVECs were exposed to 100 μM H_2_O_2_ for 1 h. Western blot assay showed that there was no significant difference in the expression of senescence-associated proteins p16 and p21 between the anti-miRNA-142-3p group and the control group in normal HUVECs. However, when cells were treated with both anti-miR-142-3P and 100 uM H_2_O_2_, protein levels of p16 and p21 were not as ‘highly elevated’ as upon treatment with anti-miR-NC + 100 uM H_2_O_2_ (Figure 4A–C). Furthermore, the SA-β-gal staining assay was performed to evaluate the degree of cellular senescence in HUVECs transfected with miRNA-142-3p. The results of the SA-β-gal staining assay showed that the percentage of SA-β-gal-positive cells was not significantly different between the HUVECs transfected with anti-miRNA-142-3p and those transfected with anti-miR-NC. Conversely, the percentage of SA-β-gal-positive cells in premature senescent HUVECs transfected with anti-miRNA-142-3p was significantly decreased compared with premature senescent HUVECs transfected with anti-miR-NC (Figure 4D,E).

### Knockdown of miRNA-142-3p improved the migration and proliferation of premature senescent HUVECs induced by H_2_O_2_

**Figure 5:**
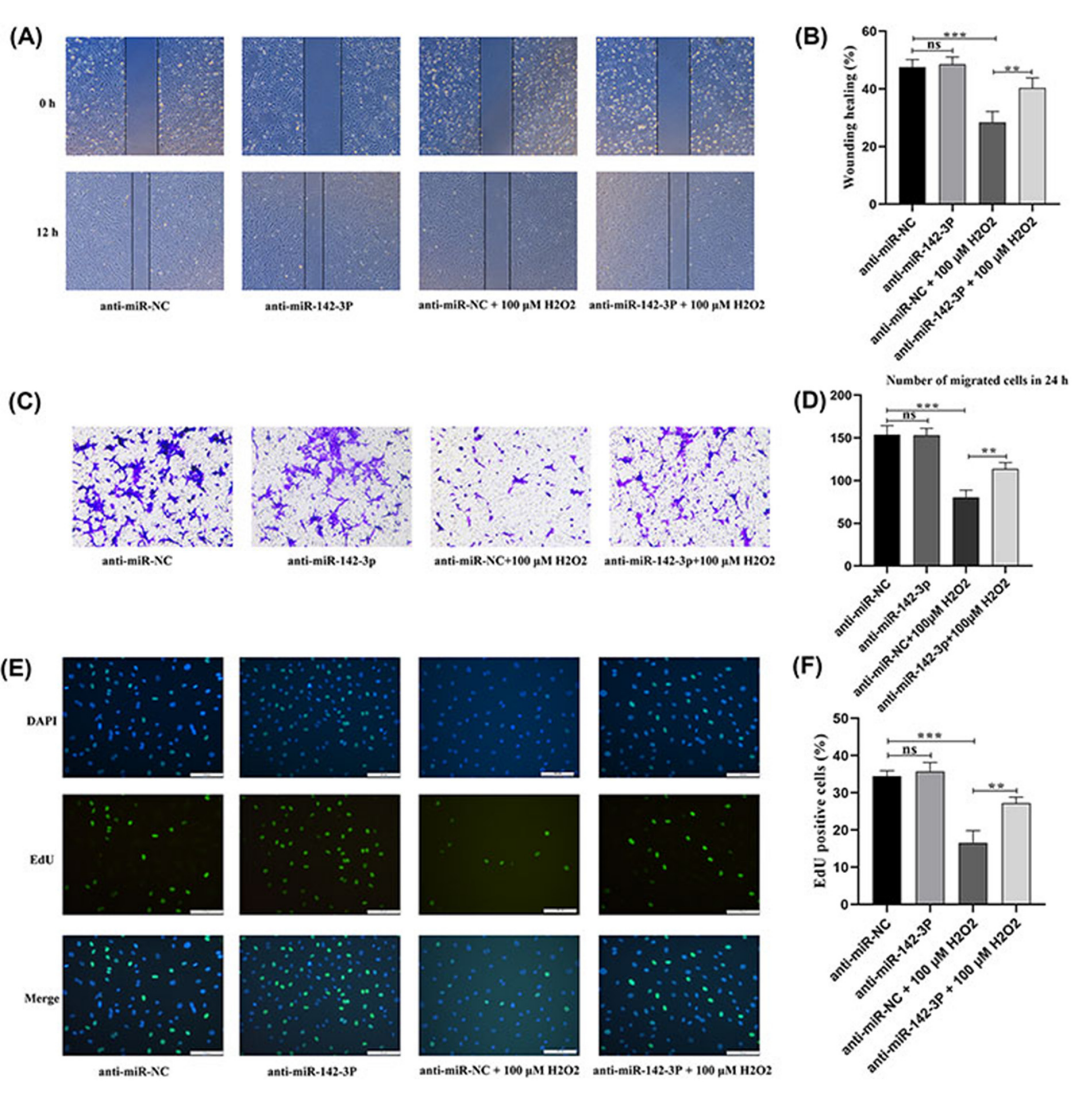
Knockdown of miR-142-3p improved the migration and proliferation ability of premature senescent HUVECs (**A,B**) Representation and statistics of cell migration in HUVECs with different miR-142-3p gene modifications in the scratch wound-healing experiment. (**C,D**) Representation and statistics of cell migration in HUVECs with different miR-142-3p gene modifications in the transwell experiment. (**E,F**) Representation and statistics of cell proliferation in HUVECs with different miR-142-3p gene modifications in the EdU staining assay. **P<0.01, ***P<0.001, HUVEC, human umbilical vein endothelial cell; ns: no significance.

Senescent cells lose their ability to divide due to permanent cyclic arrest. Therefore, the scratch wound-healing assay, transwell assay, and EdU staining assay were performed to evaluate the effects of miRNA-142-3p on the HUVECs migration, infiltration, and proliferation. The scratch wound-healing assay showed that miRNA-142-3p has no obvious effect on the migration ability of normal HUVECs but significantly inhibits the migration ability of prematurely senescent endothelial cells (Figure 5A,B). The transwell assay showed no difference in the number of infiltrated cells transfected with miRNA-142-3p inhibitor versus NC-inhibitor (Figure 5C,D). However, the number of infiltrated cells was significantly increased in premature senescent HUVECs transfected with miRNA-142-3p inhibitor compared with premature senescent HUVECs transfected with NC-inhibitor (Figure 5C,D). These results are consistent with the EdU assay results (Figure 5E,F), and suggest that inhibition of miRNA-142-3p expression can improve the proliferation ability of senescent endothelial cells. Furthermore, knockdown of miRNA-142-3p improves the migration, infiltrative, and proliferative ability of premature senescent HUVECs induced by H_2_O_2_.

### SIRT1 is a direct target of miRNA-142-3p in HUVECs

**Figure 6:**
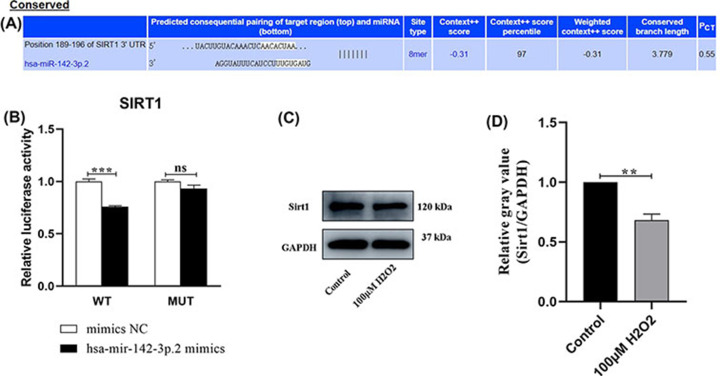
SIRT1 was a direct target of miR-142-3p in HUVECs (**A**) The binding sites between miR-142-3p and SIRT1 were predicted by TargetScan software (https://www.targetscan.org/cgi-bin/targetscan/vert_72/view_gene.cgi?rs=ENST00000212015.6&taxid=9606&members=miR-142-3p.2&showcnc=0&shownc=0&subset=1). (**B**) Luciferase assay confirmed the target relationship between miR‐142‐3p and SIRT1. SIRT1-WT binding with miR-142-3p.2 mimics significantly reduced the luciferase activity, while SIRT1-MUT could not bind with miR-142-3p and had no significant effect on luciferase activity. (**C,D**) Representation and statistics of SIRT1 protein expression in HUVECs between control and H_2_O_2_ treatment groups, revealed by Western Blot experiment; **P<0.01, ***P<0.001, HUVEC, human umbilical vein endothelial cell; ns: no significance.

Previous studies have shown that miRNA-142-3p targets SIRT1 in ovarian cells, PC12 cells, and macrophages [26–28]. Targetscan software (https://www.targetscan.org/vert_72/) predicted the specific binding site of miR-142-3p in the SIRT1 3′UTR (Figure 6A). To further explore whether miRNA-142-3p also targets SIRT1 in HUVECs, a reporter plasmid was constructed by cloning the SIRT1 3′-UTR into the pmirGLO vector Dual-luciferase miRNA Target Expression Vector (Genepharma, Shanghai, China). The SIRT1 reporter or a control plasmid (empty vector) was then co-transfected into HUVECs with the miRNA-142-3p mimic. Cells co-transfected with the mimic and the SIRT1 3′-UTR vector had significantly lower dual-luciferase activity than cells transfected with the mimic and the empty plasmid, indicating that SIRT1 is a potential target of miR-142-3p (Figure 6B). Western blot analysis revealed that the expression levels of the SIRT1 protein were significantly reduced in the premature senescence group compared to the control group (Figure 6C,D). Overall, SIRT1 is negatively related to miRNA-142-3p expression in HUVECs.

### miRNA-142-3p accelerates premature senescence by down-regulating SIRT1 expression in HUVECs

**Figure 7:**
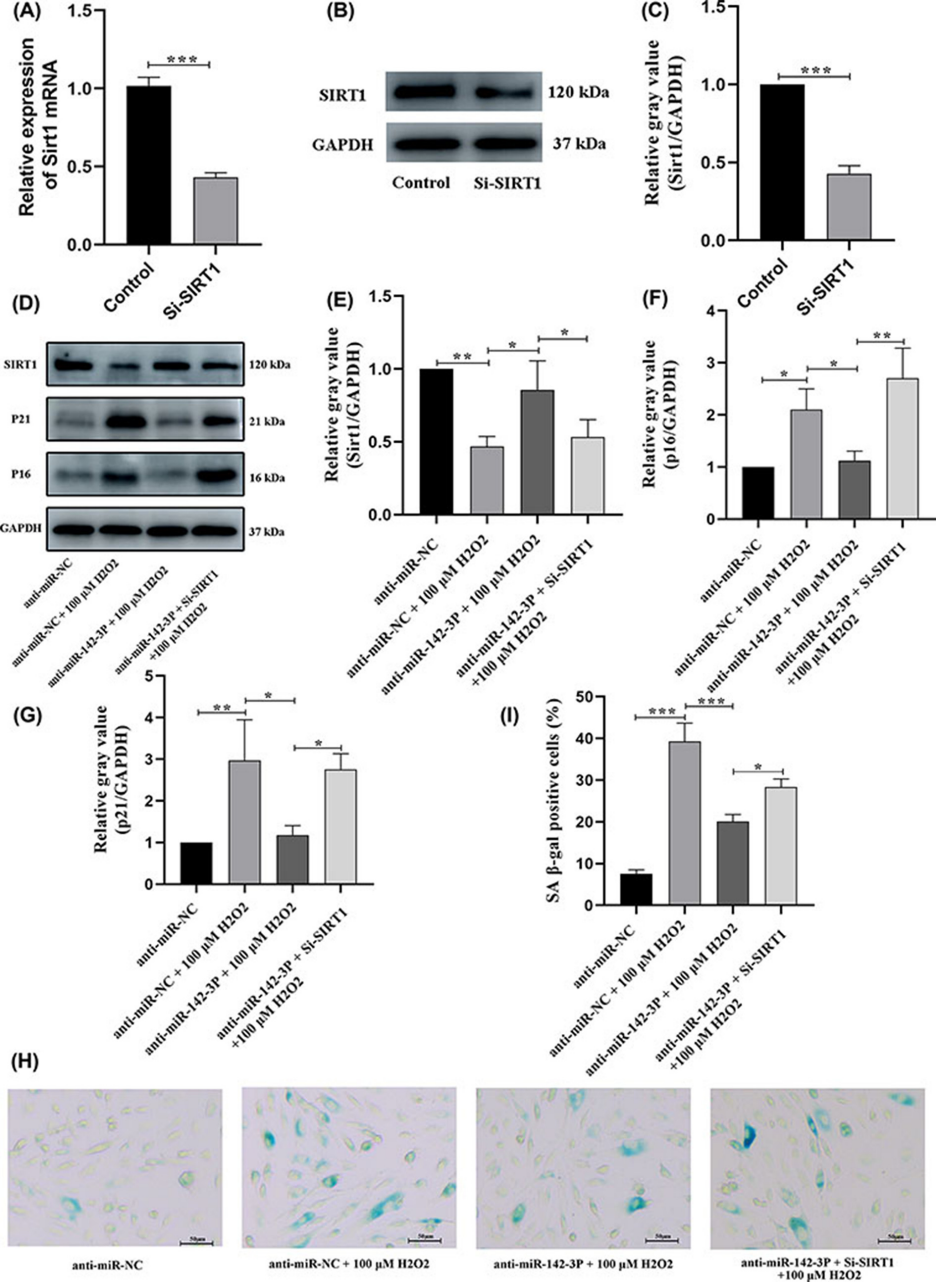
miR-142-3p accelerates premature senescence by down-regulating SIRT1 expression in HUVECs (**A**) The mRNA expression level of SIRT1 in the control and Si-SIRT1 groups was revealed by the RT-qPCR experiment. (**B,C**) Representation and statistics of SIRT1 protein relative expression level in control and Si-SIRT1 group revealed by Western Blot. (**D–G**) Representation and statistics of SIRT1, p16, and p21 protein relative expression levels between treatment groups revealed by Western Blot. (**H,I**) Representation and statistics of cell premature senescence between treatment groups revealed by SA β-gal staining assay. Senescent cells become larger and stained blue. *P<0.05, **P<0.01; ***P<0.001; HUVECs: human umbilical vein endothelial cells. SA β-gal staining assay: Senescence-associated β-galactosidase staining assay.

To further explore whether miRNA-142-3p accelerated HUVEC premature senescence via regulating SIRT1, SIRT1 mRNA and protein expression levels were determined in si-SIRT and si-NC treated HUVECs. SIRT1 mRNA and protein levels were significantly decreased in si-SIRT1 compared to si-NC HUVECs (Figure 7A–C). Compared with the anti-miR-NC group, the expression level of SIRT1 protein in the anti-miR-NC + 100 μM H_2_O_2_ group was significantly decreased. When miR-142-3p expression was inhibited via anti-miR-142-3p, and the SIRT1 expression level was reversed. Western blot assay revealed that the senescence-associated proteins p16 and p21 were also decreased. Furthermore, when si-SIRT1 was used to block the expression of SIRT1, the expression of SIRT1 was decreased while the expression of senescence-related proteins p16 and p21 were significantly increased and the degree of cell senescence was exacerbated in the anti-miR-142-3p group compared to the anti-miR-142-3p + 100 μM H_2_O_2_ group (Figure 7D–G). Cell senescence was also assessed using the SA β-gal staining assay, and results were consistent with those obtained by Western blot (Figure 7H,I). These findings suggest that miRNA-142-3p accelerates premature senescence in HUVECs by targeting SIRT1.

### miRNA-142-3p inhibited the migration and and proliferation ability of premature senescence by down-regulating SIRT1 expression in HUVECs

**Figure 8:**
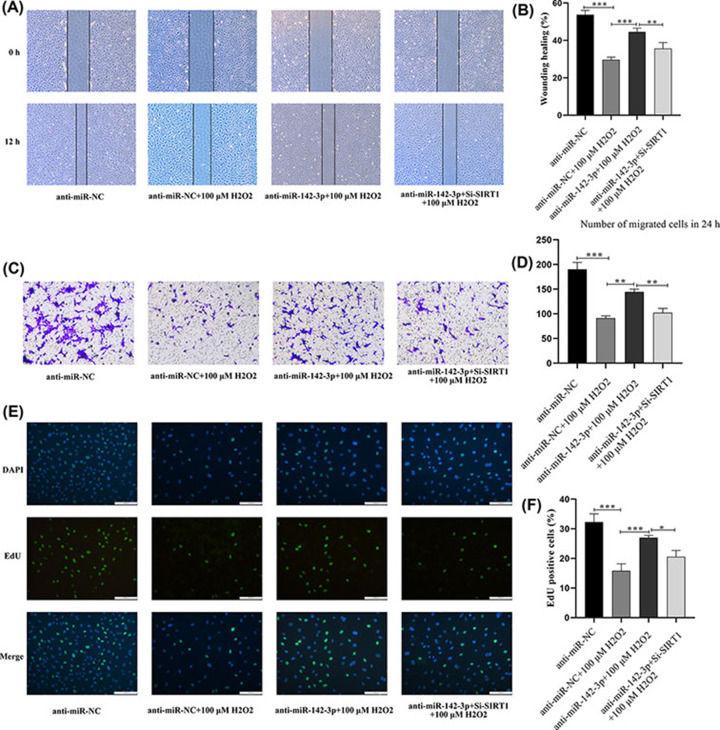
miR-142-3p inhibited the migration and proliferation ability of premature senescence by down-regulating SIRT1 expression in HUVECs (**A,B**) Representation and statistics of cell migration in HUVECs between treatment groups in the scratch wound-healing experiment. (**C,D**) Representation and statistics of cell migration in HUVECs between treatment groups in the transwell experiment. (**E,F**) Representation and statistics of cell proliferation in HUVECs between treatment groups in the EdU staining assay; *P<0.05, **P<0.01, ***P<0.001, HUVEC, human umbilical vein endothelial cell; ns: no significance.

Cell function assay was used to examine whether miRNA-142-3p affected the migration and proliferation of endothelial cells by targeting SIRT1. The cells were divided into the following groups: anti-miR-NC, anti-miR-NC + 100 μM H_2_O_2_, anti-miR-142-3p + 100 μM H_2_O_2_, and anti-miR-142-3p + si-SIRT1 + 100 μM H_2_O_2_. Cell scratch assay and transwell assay showed that the migration ability of the senescent cells was significantly lower than that of the anti-miR-NC group (Figure 8A–D). Compared with the anti-miR-NC + 100 μM H_2_O_2_ group, cell migration ability was significantly increased after inhibiting miR-142-3p expression, and this change in cell migration ability was further inhibited by inhibiting SIRT1 expression. Meanwhile, the results of the EdU cell proliferation assay (Figure 8E,F) confirmed that, compared with the anti-miR-NC group, the proliferative ability of HUVECs cells in the premature senescence group induced by H_2_O_2_ was significantly decreased. After inhibiting the expression of miR-142-3p, the migration ability of HUVECs was significantly increased. Thus, the migration ability of endothelial cells was significantly decreased by inhibiting SIRT1 expression. The above results confirmed that miR-142-3p inhibits the migration and proliferation of HUVECs by targeting and down-regulating SIRT1 expression.

## Discussion

Accumulating evidence indicates that endothelial dysfunction not only serves as a marker of vascular diseases but also plays an important role in the pathological progression of cerebrovascular events [4,29]. The vascular endothelium plays an important physiological role in vascular homeostasis by regulating vascular tone, blood flow, inflammation and immunity, and angiogenesis [30]. Several phenotypic and physiological adaptions occur during cellular senescence of vascular endothelial cells. They become flattened and enlarged with large polypoid nuclei and lose their ability to divide [7]. These changes affect cytoskeleton integrity, cell proliferation, cell migration, and angiogenesis, leading to vascular dysfunction [30,31]. microRNA has been reported to be involved in regulating the pathological process of vascular disease progression, making it a potentially good candidate for stroke intervention.

It is widely acknowledged that microRNA plays an important role in the development of human diseases [32]. microRNA is essential for post-transcriptional gene modification, as it regulated gene expression via translational inhibition and mRNA destabilization [13,33]. Accumulating studies have revealed that microRNA is involved in vascular endothelial cell senescence [21,34]. For example, miRNA-146a has been reported to alleviate endothelial cell senescence induced by oxidative stress by regulating Src [6]. The results of Li Yan demonstrated that miRNA-199a-3p accelerates the endothelial senescence induced by high glucose levels by increasing DDR1 [35]. Additionally, Shujun Yang et al. observed that overexpression of miRNA-216a promotes endothelial senescence and inflammation by targeting the Smad3/IκBα signaling pathway [36].

As a member of the microRNA family, miR-142-3p has been shown to play an important role as a tumor suppressor in several common cancer types [37–39]. For instance, Marianna Colamaio et al. proved that miRNA-142-3p inhibits the proliferation of thyroid follicular carcinoma by regulating ASH1L and MLL1 [40]. The results of Peng Liu et al. demonstrated that microRNA-142-3p overexpression inhibits colorectal cancer tumorigenesis by regulating catenin expression and inhibiting Wnt signaling activation [41]. Lu Liang et al. reported that microRNA-142-3p overexpression enhanced the drug sensitivity of breast cancer and suppressed autophagy via HMGB1 regulation [42]. These findings implied that the aberrant expression of miRNA-142-3p may be associated with progression of vascular endothelial senescence. Therefore, we aimed to determine here if miR-142-3p is involved in the regulation of prematurely senescent HUVECs. By exploring the expression levels of miRNA-142-3p in the normal HUVECs and premature senescent HUVECs, we discovered that miRNA-142-3p was highly expressed in premature senescent HUVECs. Additionally, the degree of endothelial cell senescence could be reduced by inhibiting microRNA-142-3p expression, which also improved endothelial cell migration, infiltration, and proliferation.

SIRT1 is a main histone deacetylase in mammals that plays an important role in vascular-related diseases [43]. Interestingly, SIRT1 is a direct target of miRNA-142-3p in ovarian cancer [26]. Thus, SIRT1 is a longevity gene that regulates cell metabolism and DNA repair via deacetylation of histone and p53 proteins, and it may play a role in cancer prevention or promotion [44]. Here, we used bioinformatics and luciferase reporter gene analysis to identify SIRT1 as a potential target of microRNA-142-3p in HUVECs. Weijin Zhang et al. reported that SIRT1 protects vascular endothelial cells from oxidative stress by regulating eNOS, NOX, NF-κB, SOD, and other signaling pathways [45]. SIRT1 also regulates cerebral vascular function by inhibiting nitric [46]. In our study, the expression of SIRT1 was significantly decreased in premature aging in HUVECs. However, inhibition of miRNA-142-3p expression could significantly improve SIRT1 expression in endothelial cells treated with H_2_O_2_. Moreover, inhibition of SIRT1 expression reversed the effects of miRNA-142-3p on HUVEC viability and rescued the promotion effects of miRNA-142-3p on cell senescence under oxidative stress conditions. These results suggest that miRNA-142-3p exacerbates hydrogen peroxide-induced human umbilical vein endothelial cell premature senescence by targeting SIRT1.

The underlying mechanism of miR-142-3p overexpression in HUVECs induced by H_2_O_2_ is still unclear and will be further investigated in future studies. Many long non-coding RNAs (lncRNAs) have been reported to down-regulate miRNA expression by competitively binding to miRNA. For instance, lncRNA MALAT1 inhibits inflammation and oxidative stress in injured PC12 cells induced by OGD/R by negatively regulating miRNA-142-2p [27]. lncRNA-NEAT1 exerts a cardioprotective effect by targeting the miR-142-3p/FOXO1 signaling pathway [47]. Given the interactions between miRNAs and lncRNAs, upstream lncRNAs will be a focus of future studies on the mechanism of H_2_O_2_-induced miR-142-3p overexpression.

## Conclusion

In the present study, our data revealed that a stable and reliable premature senescence model of HUVECs could be established by treating HUVECs with 100 μM H_2_O_2_ for 1h. Further investigation revealed that miRNA-142-3p, which is significantly up-regulated in premature senescent HUVECs, may promote senescence while suppressing HUVEC migration, infiltration, and proliferation, at least partially through direct targeting of SIRT1. As a result, the current study's findings indicate that miRNA-142-3p has great research potential in the pathogenesis of cerebrovascular accidents.

## Supplementary Material

Supplementary Figure

## Data Availability

The analyzed data sets generated during the study are available from the corresponding author on reasonable request.

## Abbreviations

BOD1: biorientation of chromosomes in cell division 1
ECL: enhanced chemiluminescence
ECM: endothelial cell medium
HUVEC: human umbilical vein endothelial cell
PVDF: polyvinylidene fluoride
SA-β-gal: senescence-associated β-galactosidase
SASP: senescence-associated secretory phenotype
SIRT1: silencing information regulator 2 related enzyme 1
VEZF1: vascular endothelial zinc finger 1
