# Supplementary figures and images for: miRNA-142-3p aggravates hydrogen peroxide-induced human umbilical vein endothelial cell premature senescence by targeting SIRT1
Source: Biosci Rep. 2024 May 15;44(5):BSR20231511. doi: 10.1042/BSR20231511 (PMC11096645; doi:10.1042/BSR20231511)

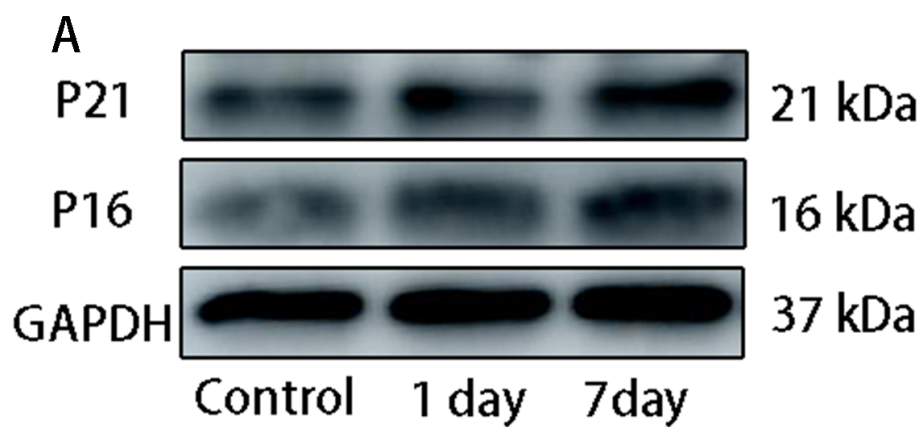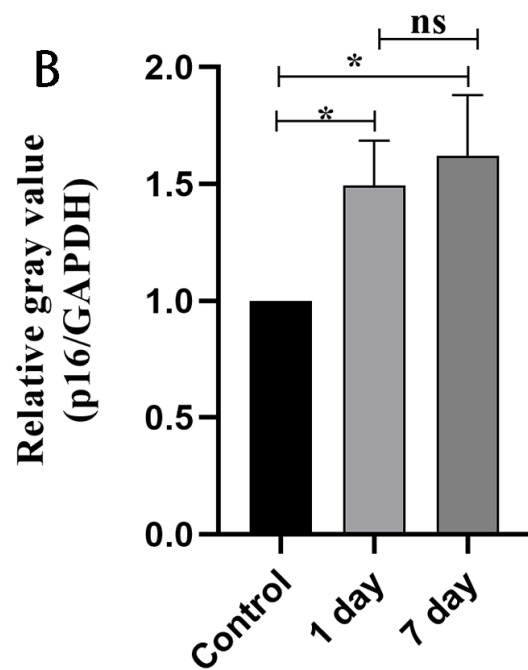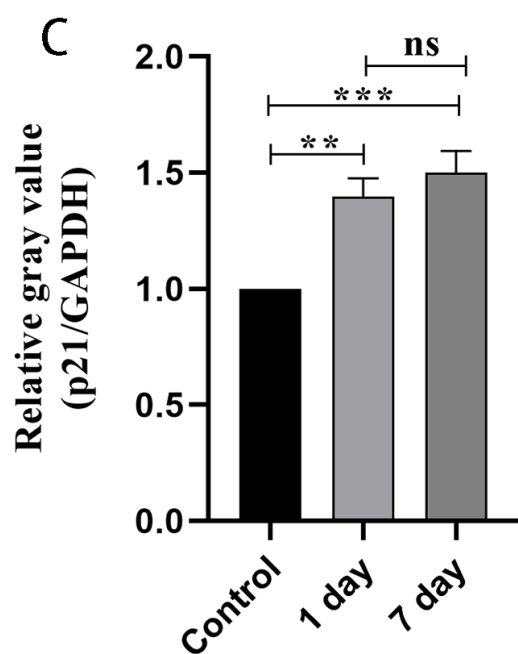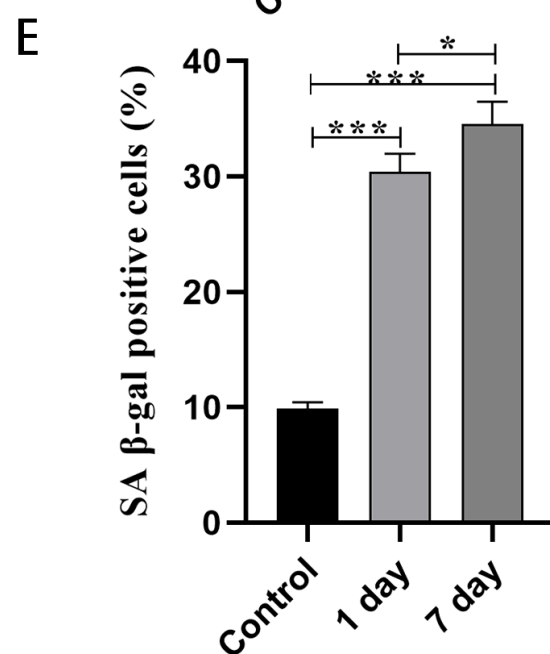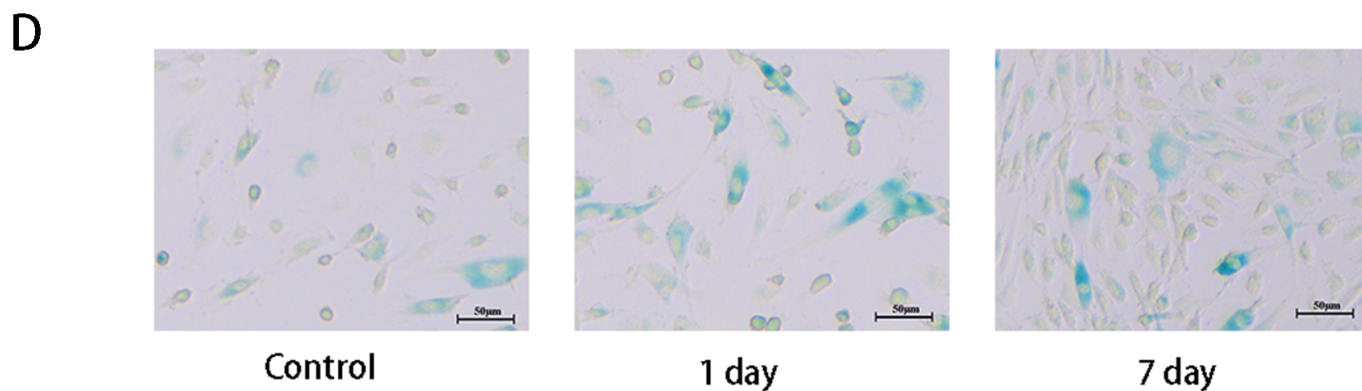

Supplement: Supplementary Figure [file BSR-2023-1511_supp.pdf]
